# Evaluation of High-Dose Antipsychotic Monitoring Within the Bellshill Community Mental Health Team

**DOI:** 10.1192/bjo.2026.11559

**Published:** 2026-06-30

**Authors:** Hayley Macpherson, Muqdad Aldar, Saba Ansari, Elizabeth Spence

**Affiliations:** NHS Lanarkshire, Glasgow, United Kingdom

## Abstract

**Aims:**

High-dose antipsychotic therapy (HDAT) refers to antipsychotic prescribing above recommended limits, either as a single drug exceeding the BNF maximum dose or combined drugs with a total dose above 100% of the maximum dose. This may be necessary for some patients due to symptom severity or treatment resistance; however, it is associated with an increased risk of side effects.

NHS Lanarkshire guidelines suggest that patients on HDAT should have regular monitoring of clinical progress, risk factors, side effects, ECGs, LFTs, U&Es, temperature and blood pressure. At present, there are no dedicated clinics in the region to monitor patients on high-dose antipsychotics.

This audit therefore aims to investigate the number of patients within the Bellshill Community Mental Health Team (CMHT) who are prescribed HDAT, and to assess whether appropriate monitoring has been carried out in line with local guidelines.

**Methods:**

The medical notes of 551 patients open to psychiatry in the Bellshill locality were reviewed to see how many were on high dose antipsychotics. The health records of the patients on high dose antipsychotics were then reviewed to see how many had appropriate monitoring in the last 6 months in line with NHS Lanarkshire guidelines.

**Results:**

There were 551 patients under Bellshill CMHT of which 366 were on antipsychotics (48%). Of the 366 patients on antipsychotics, 10 were prescribed HDAT (2.7%). None of the patients on HDAT had evidence of full appropriate monitoring within the past six months in accordance with local guidelines. Among the 10 patients receiving HDAT, 7 (70%) had appropriate bloods taken in the last 6 months, while only 1 (10%) had an ECG completed within the last 6 months.

**Conclusion:**

The findings demonstrate that high-dose antipsychotic monitoring is not being consistently carried out in Bellshill CMHT. This has prompted the expansion of the audit across Lanarkshire and underlines the need for HDAT monitoring clinics to improve patient safety and adherence to recommended standards. Further work is recommended to develop robust monitoring strategies for patients on HDAT, including the implementation ofstructured monitoring sheets, electronic alert systems, and dedicated clinic appointments for ongoing assessment.

